# Felt Splints

**Published:** 1853-12

**Authors:** Frank H. Hamilton


### ART. V.—*Felt Splints.* By Frank H. Hamilton, M. D.

Dear Doctor,—Some years ago, I think in 1845, Felt Splints were brought to me by an agent of the manufacturer. The felt was sold in sheets, and also in pieces, modeled so as to be readily adapted to the form of the limbs. In some respects it was superior to gutta percha, and I am inclined to think that on the whole it was the best splint ever used. I cannot learn that these splints are now manufactured in any part of the United States, and I will therefore inclose you the recipe for making them, which was kindly given me by the agent, and which I have frequently used myself:

### *Felt Splints.*

Dissolve three pounds of gum shellac in two quarts of alcohol. It should be dissolved in a tin vessel, furnished with a tight cover to prevent evaporation. Spread a piece of old or new woolen cloth on a board, and with a clean brush saturate both sides of the cloth with the solution. Hang it up

---

until it is thoroughly dried. Lay it again upon the board and apply a second coat of the solution to one side only of the cloth. Dry again, and apply a third coat to the same side. There will now be three successive layers upon one side and one on the opposite. While the last coat is yet fresh, fold the cloth so that the side having three coats shall be applied to itself. Now with a hot flat-iron, smooth and press the surfaces together. When it is cold, a slight rubbing with sand paper makes it fit for use.

It becomes a firm, almost unyielding board, but exposure to a moderate heat will make it pliant, so that it can easily and accurately be adapted to any surface.

